# Mechanochemical Synthesis and Electron Crystallography Characterization of van der Waals Lanthanoid 2D Metal–Organic Frameworks

**DOI:** 10.1021/acs.inorgchem.5c01592

**Published:** 2025-06-27

**Authors:** Franco Lorenzo, Chrysanthi Katsavou, Kevin Parada Rolán, Sara Dias, Helena Fernández Cortés, Javier Collado, Francisco Javier Chichón, Rocio Arranz, César Santiago, E. Carolina Sañudo

**Affiliations:** † Departament de Química Inorgànica i Orgànica, 16724 Universitat de Barcelona, C/Martí i Franqués 1-11, Barcelona 08028, Spain; ‡ IN2UB Institut de Nanociència i Nanotecnologia, Universitat de Barcelona, C/Martí i Franqués 1-11, Barcelona 08028, Spain; § CryoEM Facility, Department of Macromolecules Structure at CNB, CSIC, C/Darwin 3, Campus Universidad Autónoma de Madrid, 28049 Madrid, Spain

## Abstract

Mechanochemical synthesis provides access to clean, effective, and rapid synthesis of 2D van der Waals MOFs [Ln­(MeCOO)­(PhCOO)_2_] (**1Eu**, **2Eu**, **2Tb**). The method also allows easy access to heterometallic analogue **2LaTb**. All samples are obtained as nanosized crystals, and **1Eu** has been characterized by electron crystallography (3D-ED). Thus, mechanochemistry and 3D-ED is a winning combination for the clean synthesis of 2D MOFs that can be upscaled to multigram synthesis.

## Graphical abstract

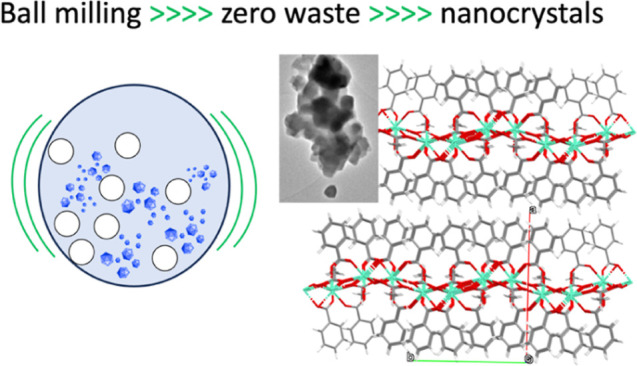

## Introduction

A mechanochemical reaction is defined by IUPAC as a reaction that is initiated and sustained by the direct absorption of mechanical energy.^1^ The mechanical force can be provided by impact, grinding, milling, compression, friction, or stretching. Mechanochemical synthesis (or mechanosynthesis) is of great interest to the chemical industry since it provides opportunities for chemical reactions in quantitative yield and avoids generation (and treatment) of chemical waste. Additionally, scale-up to multigram synthesis is easily done for mechanochemical processes. This makes mechanochemistry a great tool for the chemical industry to meet UN’s sustainable development goals.^2^ Translation to industrial synthesis from the batch ball mill synthesis discussed here can be explored with continuous screw mechanochemical synthesis.^3^

Due to this, mechanochemical synthesis is now gaining new attention. Sustainable development, waste management, and atomic economy in chemical reactions are nowadays priorities for the chemical industry and for any chemical laboratory. Mechanochemical systems provide a solvent-free reaction method that can be exploited to obtain quantitative yields. Mechanochemistry has been used in the last years in the fields of organic synthesis,^4–6^ coordination chemistry,^7,8^ main group chemistry,^9^ or supramolecular chemistry.^10^ Recently, the shear forces that happen in a ball-mill have been applied to attain new ice phases with density similar to that of liquid water.^11^ The most used methods to perform mechanochemistry were reviewed in detail in 2020.^12^ In general, the easiest way to perform mechanochemical synthesis is mortar-and-pestle grinding, which is done manually, but automated modes of providing the mechanical energy are more efficient and reproducible. Mechanochemical syntheses are thus usually performed in ball-mills that can provide a controlled amount of mechanical energy. Usually the reagents are introduced into a milling jar along with one or several milling balls. The material of the jar and balls should be able to withstand the reagents without reacting or being corroded by the products of the reaction. Typical jar and ball materials are Teflon, stainless steel, or zirconia. Nowadays, PMMA transparent jars allow for Raman monitoring of the mechanochemical reaction in real time.^13^ Additionally, additives such as agglutinating media or a small amount of solvent can be used to assist or direct the mechanical reaction. The characterization of obtained products relies mostly on powder X-ray diffraction pattern analysis; however, for MOFs, it is not always possible to achieve a full structure from PXRD. One drawback of mechanochemical synthesis can be the obtention of amorphous materials. Amorphous materials might require a thermal treatment or annealing after the mechanochemical synthesis to attain a crystalline product.^14^ In the reactions discussed here, the products are nanocrystalline. We combine mechanochemical synthesis with PXRD, SEM spectroscopy, and electron diffraction crystallography to characterize the obtained materials. Electron diffraction crystallography, also called 3D-ED or microED, is a technique that is gaining force among the crystallography community,^15^ in particular for MOFs^16^ and COFs.^17^ Micro-ED allows us to determine crystalline structures for nanometer-sized crystals^18^ using a continuous rotation stage on a TEM microscope. Thus, it is an ideal companion to mechanochemical synthesis.

The photoluminescent and magnetic properties of lanthanoid ions make them of great interest for applications such as sensors, quantum computing, or information storage. The integration of lanthanoid ions in MOFs is thus a logical step to expand the applications of MOFs. In particular, research on 2D-MOFs based on lanthanoids is interesting since these materials can provide ordered arrays of single-molecule magnets (SMMs) for information storage or arrays of qubits for quantum information processing technologies. 2D MOF arrays of qubits can be designed so as to control dipolar interactions and magnetic dilution, which impact quantum decoherence, and to integrate qubits into devices.^19^ Additionally, Ln­(III) 2D MOFs offer processable materials for on-surface magnetic refrigeration leveraging the magnetocaloric effect (MCE),^20,21^ metallic conductivity when combined with the proper ligands,^22^ and multifunctional materials combining magnetism and luminescence for novel magneto-optical applications.^23–28^

In 2021, we reported 2D MOFs of formula [Ln­(MeCOO)­(PhCOO)_2_] (Ln = Dy,^25^ Tb,^29^ Gd^30^) that were prepared using a microwave reactor from hydrated lanthanoid acetate and benzoic acid, with acetic acid as a byproduct. In 2023, we extended the study to Eu and TbEu heterometallic complexes with outstanding luminescent properties.^28^ In this reaction, the only byproduct is a volatile organic compound (acetic acid); thus, we decided to test this reaction using mechanochemical synthesis. The hypothesis is that reactions where the only byproducts are volatile organic compounds or H_2_O are ideal for mechanochemical synthesis. We also hypothesize that using mechanochemical synthesis, quantitative yields and scalable reactions will be possible. The results applied to the synthesis of 2D MOFs are presented here.

## Discussion of Results

We studied two methods to perform mechanochemical synthesis: manual mortar-and-pestle grinding (method **1**) and ball-milling (method **2**). To prepare this material, hydrated lanthanoid acetate is reacted with two equivalents of benzoic acid. The 2D MOF of formula [Ln­(MeCOO)­(PhCOO)_2_] (***n*Ln**, *n* = 1, 2 depending on the synthesis method and Ln = Eu, Tb) can be obtained as a pure phase from the mechanochemical reaction by methods **1** and **2**. The byproduct in this reaction is acetic acid: effective removal of this byproduct is a crucial point. In these materials, the lanthanoid ion is in the oxidation state +3, and given the three carboxylate ligands per lanthanoid ion, the 2D layers are neutral.

The reaction consists of a coordination reaction coupled with proton exchange where two acetate ligands (MeCOOH p*K*_a_ = 4.76) are replaced by the conjugated base of the benzoic acid (p*K*_a_ = 4.20) forming acetic acid, Ln­(MeCOO)_3_(s) + 2PhCOOH(s) = [Ln­(MeCOO)­(PhCOO)_2_](s) + 2MeCOOH (g).

The manual mortar-and-paste synthesis (method **1**) consists of grinding the mixture of reagents manually for 20 to 220 min with rest intervals, as many as needed by the operator. For total reagent quantities of circa. 250 mg, reactions times varied between operators from 20 to 220 min; thus, these methods depending very much on the physical strength of the operator and reaction times are not reproducible. In this kind of synthesis, we add 1 drop of MeCN/MeOH after every rest interval of 5 min as mixing medium. The chosen solvent mixture is the one used in the microwave-assisted synthesis of the 2D MOFs. The process could be done without the addition of solvent, but safety measures according to local risk management protocols must be used due to the formation of extremely thin powders. During the manual grinding, acetic acid fumes are effectively expelled from the reaction mixture, so this process is performed on a fume-hood. IR spectra for **1Eu** at different grinding times are shown in the Supporting Information Figure S1. IR spectra were taken at regular intervals of grinding to follow the reaction, and the characteristic C=O stretching peak at 1683 cm^–1^ from benzoic acid clearly disappears as the reaction advances. As for the mixture of reagents, the region between 1300 to 1600 cm^–1^ exhibits also very noticeable changes: the two main peaks for coordinated carboxylate groups appear as the reaction advances to completion, marked with dashed red lines in Supporting Information Figure S1. The reaction was also controlled by PXRD of the final powders (Figure 1). Ball milling (method **2**) was performed in an Eppendorf vial using eight 5 mm zirconia balls in a Retsch Mixer Mill 400 apparatus (**2Eu** and **2Tb**). The container must be opened at short intervals to evacuate the acetic acid. The milling is stopped, and the container opened several times to vent the acetic acid. Without venting, the reaction does not reach completeness, as evidenced by the PXRD pattern of **2Eu** (no venting) (Figure 1) and by IR spectra that show the characteristic peaks of a free carboxylic acid group. Automation of the mechanochemical system is desirable to avoid uncertainties in reaction time, and we find that ball-milling works with reproducible reaction times, as opposed to manual mortar-and-pestle.

**Figure 1:**
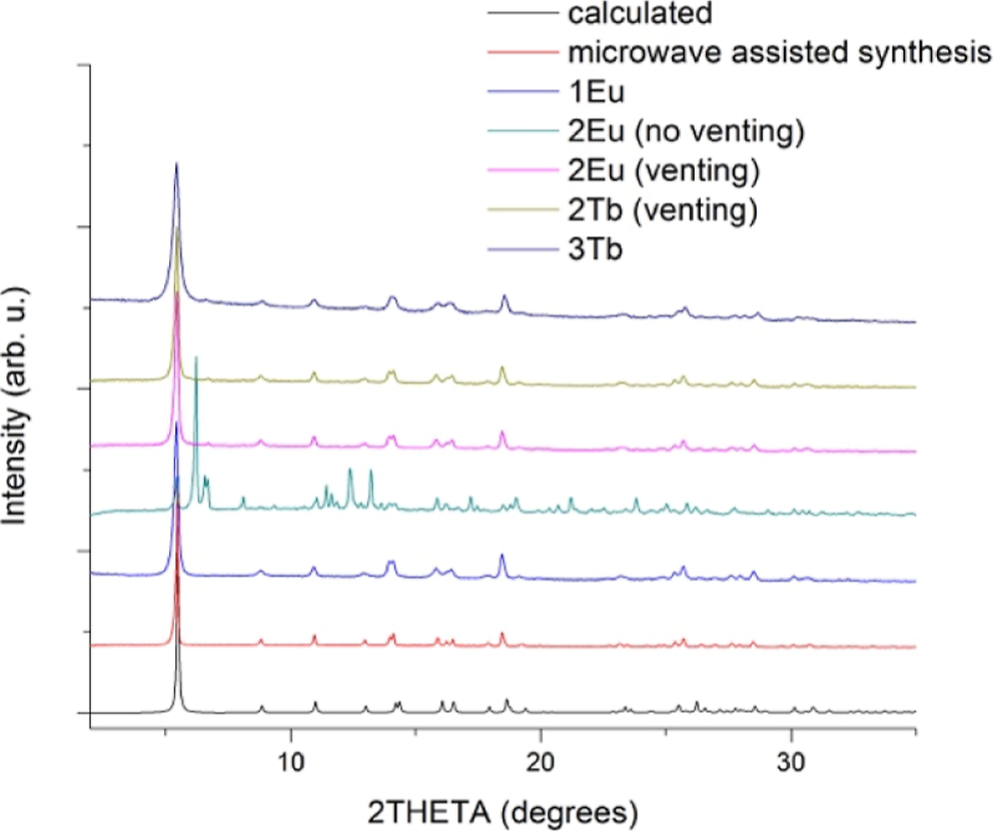
PXRD of 2D MOFs prepared by grinding methods **1Eu** (manual mortar and pestle), **2Eu**, **2Tb**, and **2LaTb** (ball-mill). (Shaker) compared to the calculated PXRD pattern from SCXRD and experimental pattern for [Eu­(MeCOO)­(PhCOO)_2_] prepared by microwave-assisted synthesis.

All products were examined by PXRD, and the patterns were compared to the calculated diffractogram of [Eu­(MeCOO)­(PhCOO)_2_] crystals obtained from the microwave reaction (microwave assisted synthesis trace in Figure 1) and the calculated pattern from the single-crystal X-ray diffraction data (calculated trace in Figure 1).^28^

[Ln­(MeCOO)­(PhCOO)_2_] (Ln = Dy, Tb, Eu, Gd) 2D MOFs are isostructural, and their PXRD patterns have a characteristic *hkl* = 100 reflection at 5.6°, related to the interlayer distance of 1.6 nm (parameter a of the unit cell). Our results show that the two mechanochemical methods **1**, **2** (manual mortar-and-pestle grinding, ball-milling) result in nanocrystalline powders, with IR spectra and PXRD patterns consistent with [Ln­(MeCOO)­(PhCOO)_2_] (**1Eu**, **2Ln**, Ln = Eu, Tb). The PXRD data are shown in Figure 1. For **2Eu** and **2Tb**, the room-temperature PXRD patterns were indexed using Expo software. Unit cells consistent with **2Eu** and **2Tb** were obtained, and the comparison between calculated and experimental patterns is collected in Supporting Information Figure S2. The first reflection, *hkl* = 100, is clearly broadened, in particular for **1Eu**. By using the Scherrer equation for the 100 reflection, the nanocrystallite size can be estimated in the nanometer range, ca. 50 nm for **2Eu** and 49 nm for **2Tb**. For **1Eu**, the PXRD pattern shows a very broad 100 peak, which indicates even smaller crystallite size. Thus, the nanocrystallites produced by ball milling are good candidates for structure elucidation using electron diffraction. To confirm the nm size of the crystallites, we dispersed 1 mg of crystals by sonication in 10 mL of isopropanol for 30 min. A clear dispersion is obtained that shows Tyndall effect, indicating that the nanometer-sized flakes or monolayers are dispersed in the solvent. The TEM images of sample **2Eu** (Figure 2) show aggregates of nanocrystals as well as isolated nanocrystals suitable for microED.

**Figure 2:**
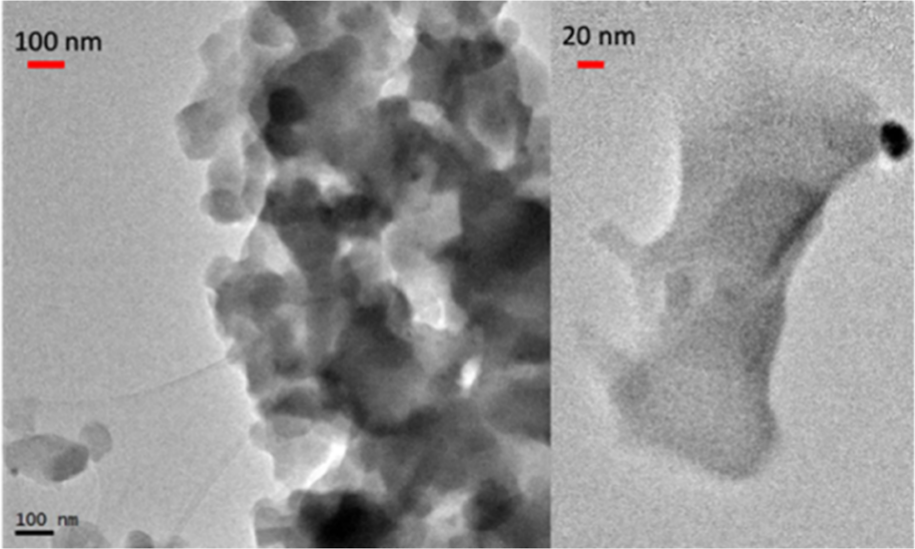
TEM images of **2Eu**, showing several nanocrystals in the left image and a very thin flake in the right image.

The nanocrystals, obtained through the aforementioned methods, were placed on a lacey carbon grid for a MicroED electron diffraction experiment conducted at the CryoEM facility of the Department of Macromolecule Structures at the CNB–CSIC, Madrid. This was carried out using a Thermo Scientific Talos Arctica 200 kV transmission electron microscope equipped with a Ceta-D camera. Diffraction images were collected using continuous rotation in a semiautomated mode with SerialEM and EPU-D software. The data were treated with the XDS program, which is integrated in the XDSGUI suite, and the structure was solved using SHELXt. A monoclinic *P*2­(1)/*c* unit cell was obtained. The crystal structure of **2Eu** shares structural parameters with [Eu­(MeCOO)­(PhCOO)_2_], with the same unit cell space group and packing. The comparison of cell parameters (SCXRD-microED) led to Δ*a* = 0.241 Å, Δ*b* = 0.139 Å, Δ*c* = 0.212 Å, Δγ = 1.21°, and Δ*V* = 69 Å^3^.^28^ As expected, 2D puckered Eu-acetato layers with benzoate ligands in a *syn*,*syn* coordination mode above and below the layer pile in the *a*-direction of the unit cell. Between layers, there are no solvent molecules, and the only interaction is van der Waals forces. The MicroED structure is shown in Figure 3.

**Figure 3:**
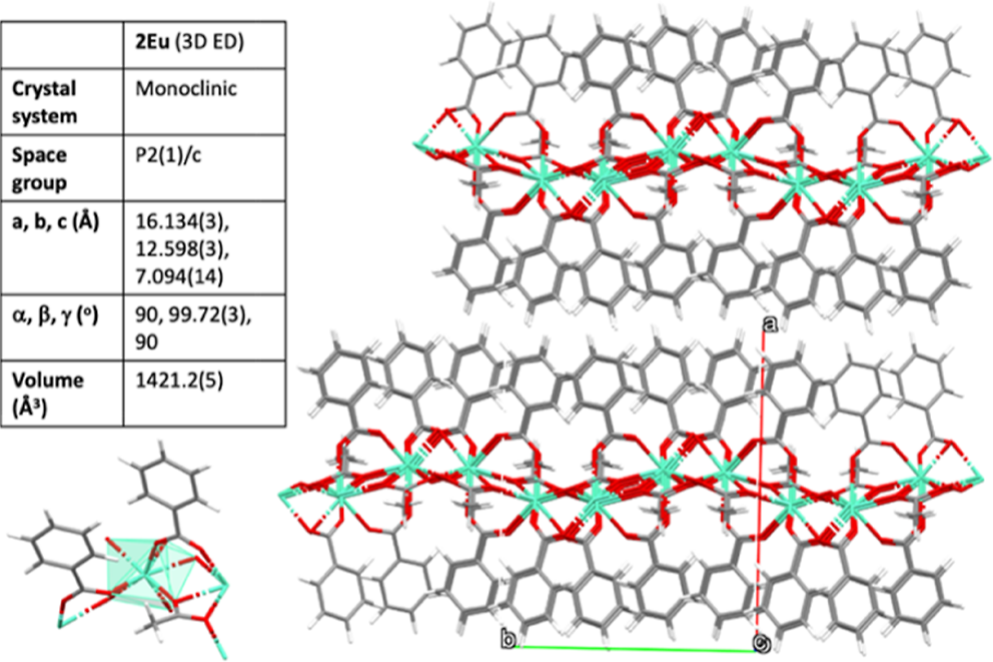
Crystal structure of **2Eu** obtained by microED. The inset table contains the unit cell parameters. Eu: green; O: red; C: gray; H; white.

The mechanochemical synthesis seems to be perfect for the preparation of heterometallic species. In the past, LaDy and EuTb analogues were prepared by using microwave-assisted synthesis. The sample **2LaTb** (with 50% Tb and 50% La) was prepared by ball milling in a Retsch MM400 ball mill. The PXRD pattern shows the expected peaks for the known structure of [La_0.5_Tb_0.5_(MeCOO)­(PhCOO)_2_], here called **2LaTb**. Semiquantitative EDS analysis shows the ratio Tb/La of 0.95 (see Supporting Information Figure S3 for SEM images). Access to heterometallic materials is very important for some applications; in particular, access to lanthanum-Ln complexes is of great interest for magnetic dilution applications. La is the largest lanthanoid, with *r*(La) – *r*(Tb) = 11 pm; thus, introduction of La can introduce some strain in the structure. Here, we show that mechanochemical synthesis can give easy efficient access to heterometallic compounds, even with large radii differences as in the case of **2LaTb**.

Figure 4 shows the emission spectra for **2Eu** and **2Tb** upon excitation at 280 nm. The emission spectrum for **2Eu** shows the expected Eu^3+^ transitions ^5^D_0_ → ^7^F_*J*_ (*J* = 0–6) and is dominated by the transition ^5^D_0_ → ^7^F_2_ centered at 617.7 nm. The emission spectrum of **2Tb** exhibits four characteristic bands that correspond to the ^5^D_4_ → ^7^F_*J*_ (*J* = 6, 5, 4, 3) transitions to the ground state multiplet of the Tb­(III) ion, dominated by the ^5^D_4_ → ^7^F_5_ transition at 546 nm. The photoluminescence characterization of **2Eu** and **2Tb** shows that their luminescent properties of the materials are intact and match those reported for the Tb and Eu materials.^28,29^

**Figure 4:**
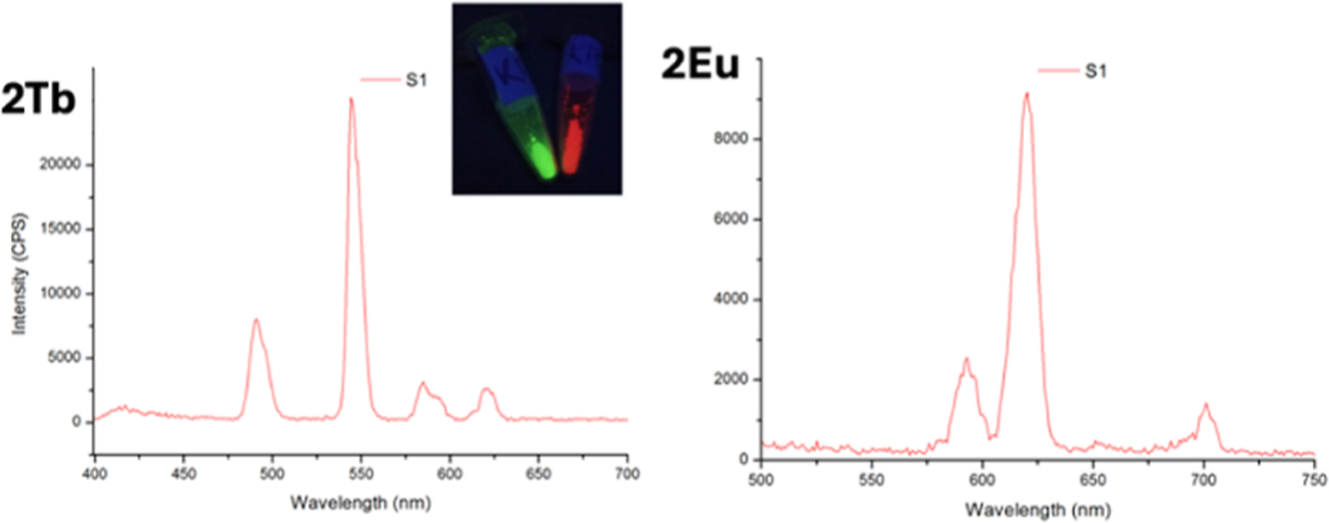
Emission spectra of **2Tb** and **2Eu** upon excitation with light of 280 nm. The inset shows a photograph of both samples (green **2Tb**, red **2Eu**) under UV light.

Further characterization included TGA and DSC in the room temperature to 350 °C temperature range. Results are shown in Supporting Information Figure S4. A sharp melting point at 284 °C is present for **2Eu** consistent with the melting point expected for this material. For **2Tb**, the DSC and TGA show the presence of an amount of unreacted starting materials, PhCOOH and hydrated Tb acetate, along with the expected melting point for [Tb­(MeCOO)­(PhCOO)_2_] at 257 °C. At temperatures above 300 °C, the materials decompose.

## Conclusions

In summary, solvent-assisted mechanochemistry is a very useful method to access large amounts of van der Waals 2D MOFs of formula [Ln­(MeCOO)­(PhCOO)_2_] (**1Eu**, **2Eu**, **2Tb**, **2LaTb**) using two different methods: **1**: manual grinding and **2**: ball milling. Both methods provide easily scalable reactions and quantitative yields, even though sample recovery can reduce the effective yield of the recovered material. From the two methods, easily implemented in any research laboratory, ball milling is the most time-efficient and reproducible in terms of reaction times and nanocrystallite size. Mechanochemical synthesis is an efficient, clean, and cheap reaction method that grants access to homometallic and heterometallic materials in quantitative yields and multigram quantities. Scaling up of chemical reactions is a very important step for any application; thus, going from a few milligrams of crystalline material for every reaction to a gram or more of nanocrystalline product is of utmost importance to assess properties of the prepared materials and properly test their applications. For example, the sensing properties of the Tb and Eu MOFs reported here vs dopamine sensing can now be tested since the materials can be easily prepared in large amounts. Access to precisely controlled heterometallic stoichiometries is particularly relevant for some applications like anticounterfeit inks, pulsed EPR experiments in magnetically diluted samples for quantum computing, or magnetic dilution of single-molecule magnets that require the preparation of heterometallic species. Furthermore, we combine mechanochemical synthesis with PRXD and MicroED crystallography to fully access structural characterization of the prepared materials as we demonstrate with **2Eu**. This is a powerful combination that, in our opinion, will lead to new successes in mechanochemical synthesis.

## Experimental Section

All reagents were purchased from commercial sources and used as received.

### [Eu­(MeCOO)­(PhCOO)_2_] **1Eu**

0.26 mmol amount of hydrated Eu­(MeCOO)_3_ and 0.52 mmol PhCOOH were placed in an agate mortar. Reagents were ground manually for 25 min. Every 5 min, a drop of 1:1 MeCN/MeOH was added before resuming grinding.

### [Ln­(MeCOO)­(PhCOO)_2_] **2Eu**, **2Tb**, **2LaTb**

0.30 mmol amount of the corresponding hydrated lanthanide acetates (for **2LaTb**, 0.15 mmol La and 0.15 mmol Tb acetate) and 0.60 mmol of PhCOOH were placed in a 5 mL Eppendorf vial with eight 5 mm zirconia balls. The Eppendorf is placed in a Teflon adapter in a Retsch Mixer Mill 400. Grinding time is 55 min at 15 Hz for **2Eu**, 35 min for **2Tb**, and 110 min for **2LaTb**. Ten grinding cycles of 1 min are applied, and as many 5 min cycles after that as necessary. After each cycle, the Eppendorf is open to vent the acetic acid, and a drop of solvent (1:1 MeOH/MeCN) is added before grinding is resumed. The reaction is monitored by IR. To recover the product, 3 mL of acetone are added to the Eppendorf, after sonication for 5 min, the solid is filtered and air-dried.

IR (is7 Nicolet ATR) and photoluminescence (Horiba Jobin Nanolog spectrophotometer) were performed on the Inorganic Chemistry Section facilities at UB.

PXRD, TGA/DSC, TEM, and SEM experiments were performed at the Scientific and Technological Centers (CCiTUB), Universitat de Barcelona. Micro-ED was performed at the CryoEM Facility at CNB, CSIC (see Supporting Information for full data collection details). Cif file of **2Eu** is available free of charge at the Cambridge Crystallographic Database with deposition code 2420395 (https://www.ccdc.cam.ac.uk/structures/). Supporting Information for this article is available online; it contains microED experimental details and further characterization (IR, PXRD, SEM, and TGA). Raw data are available from the corresponding author upon request.

## Supplementary Material



## Notes

Methods and additional characterization (PDF)
