# Supplementary material for: Mechanochemical Synthesis and Electron Crystallography Characterization of van der Waals Lanthanoid 2D Metal–Organic Frameworks
Source: Inorg Chem. 2025 Jun 27;64(27):13824–9. doi: 10.1021/acs.inorgchem.5c01592 (PMC12265042; doi:10.1021/acs.inorgchem.5c01592)

Mechanochemical synthesis and electron crystallography characterization of Van der Waals lanthanoid 2D metal organic frameworks

*Franco Lorenzo,[a] Chrysanthi Katsavou,[a] Kevin Parada Rolán,[a] Sara Dias,[a] Helena Fernández Cortés,[a] Javier Collado, [c] Francisco Javier Chichón, [c] Rocio Arranz, [c] César Santiago [c], E. Carolina Sañudo[a,b]**

[a,*] Prof. E. C. Sañudo, PhD, F. Lorenzo, S. Dias, MSc, K. Parada Rolán, MSc, H. Fernández Cortés, BSc, C. Katsavou, BSc
Departament de Química Inorgànica i Orgànica
Universitat de Barcelona
C/Martí i Franqués 1-11, 08028 Barcelona, Spain
E-mail: esanudo@ub.edu
[b] Prof. E. C. Sañudo, PhD, IN2UB Institut de Nanociència i Nanotecnologia, Universitat de Barcelona
C/Martí i Franqués 1-11, 08028 Barcelona, Spain
[c] Dr. C. Santiago, Dr. F. J. ChiChón, Dr. R. Arranz, J. Collado
CryoEM Facility. Department of Macromolecules Structure at CNB, CSIC. C/ Darwin 3. Campus Universidad Autónoma 28049, Madrid

Contents

ESI1-Methods

ESI2- Additional Characterization

Figure S1. IR spectra of **1Eu** 2D MOFs prepared by grinding method 1 (manual mortar and pestle) at different grinding times. The solid line indicates the C=O stretching of benzoic acid peak at 1683 cm-1, the peak disappears as the reaction advances towards completion. The dashed red lines indicate the two main peaks for coordinated carboxylate groups, that appear as the reaction advances to completion.
Figure S2. PXRD patterns for 2Eu and 2Tb, indexed using the software Expo2014. The calculated trace (red) is subtracted from the experimental trace.
Figure S3. SEM images and La and Tb mapping analysis for **2LaTb**.
Figure S4. Thermogravimetric analysis (TGA) and differential scanning calorimetry (DSC) for **2Tb** and **2Eu**.

ESI1- Methods

MicroED sample preparation and data collection

The grids utilized in this experiment were prepared according to the following procedure: Initially, the grids were clipped at room temperature to prevent the accumulation of sample residues at the edges from deforming the grid during the clipping process. The grids were then treated with a floating carbon layer, which was applied using a pipette.
A modest quantity of the desiccated sample was pulverised between two glass slides, and the resulting powder was deposited onto the grids. Any excess sample was removed by gently tapping the grid in order to ensure an even distribution.
Once prepared, the grids were directly immersed in liquid nitrogen and subsequently loaded into the microscope for analysis.

The grids were analysed using a Thermo Scientific™ Talos™ Arctica™ transmission electron microscope, operated at 200 kV and equipped with a Ceta-D detector. Data acquisition was performed with an electron beam wavelength of 0.025082 Å. Furthermore, a semi-automated search was conducted to identify diffracting crystals along the grating using the SerialEM software (version 4.1). Subsequently, suitable crystals were selected and collected using the EPUD software (version 1.17.0.6389). Data collection was conducted with a stage tilt of 100° (incremented by 1° per step) and an exposure time of 0.5 seconds per frame, resulting in a total dose of 1.97 e/Å^{2}s applied to each crystal.

ESI2- Additional Characterization

Figure S1. IR spectra of **1Eu** 2D MOFs prepared by grinding method 1 (manual mortar and pestle) at different grinding times. The solid line indicates the C=O stretching of benzoic acid peak at 1683 cm^{-1}, the peak disappears as the reaction advances towards completion. The dashed red lines indicate the two main peaks for coordinated carboxylate groups, that appear as the reaction advances to completion.

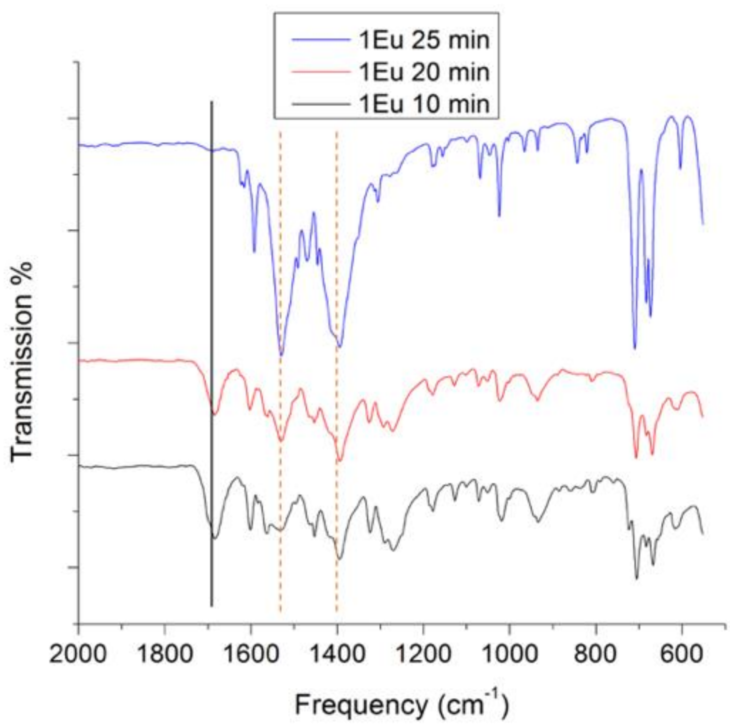

Figure S2. PXRD patterns for **2Eu**, **2Tb** and **2LaTb**, indexed using the software Expo2014. The calculated trace (red) is subtracted from the experimental trace. The PXRD patterns were obtained at room temperature.

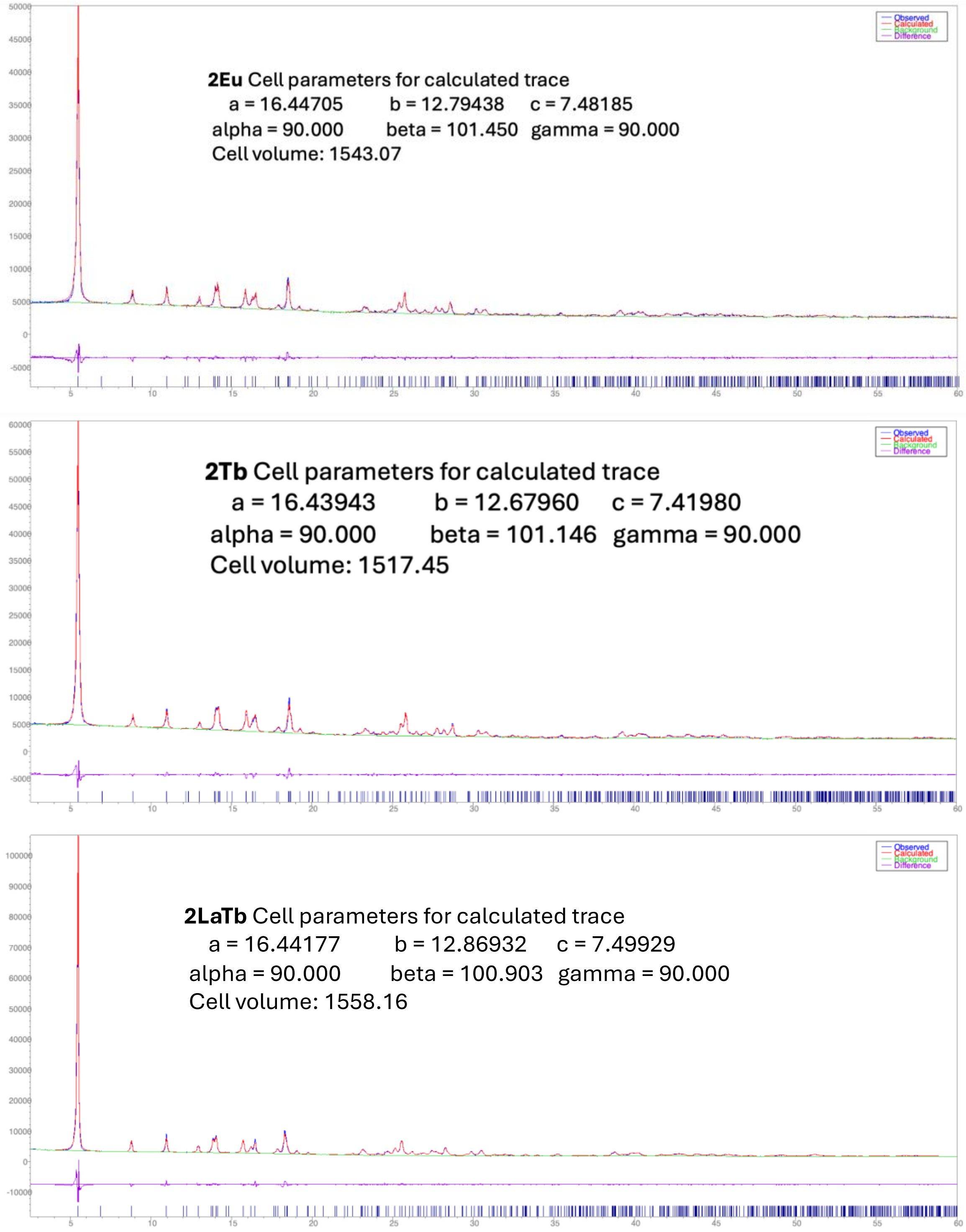

Figure S3. SEM images for **2Eu**, **2Tb** and **2LaTb**. La and Tb mapping analysis for **2LaTb**.

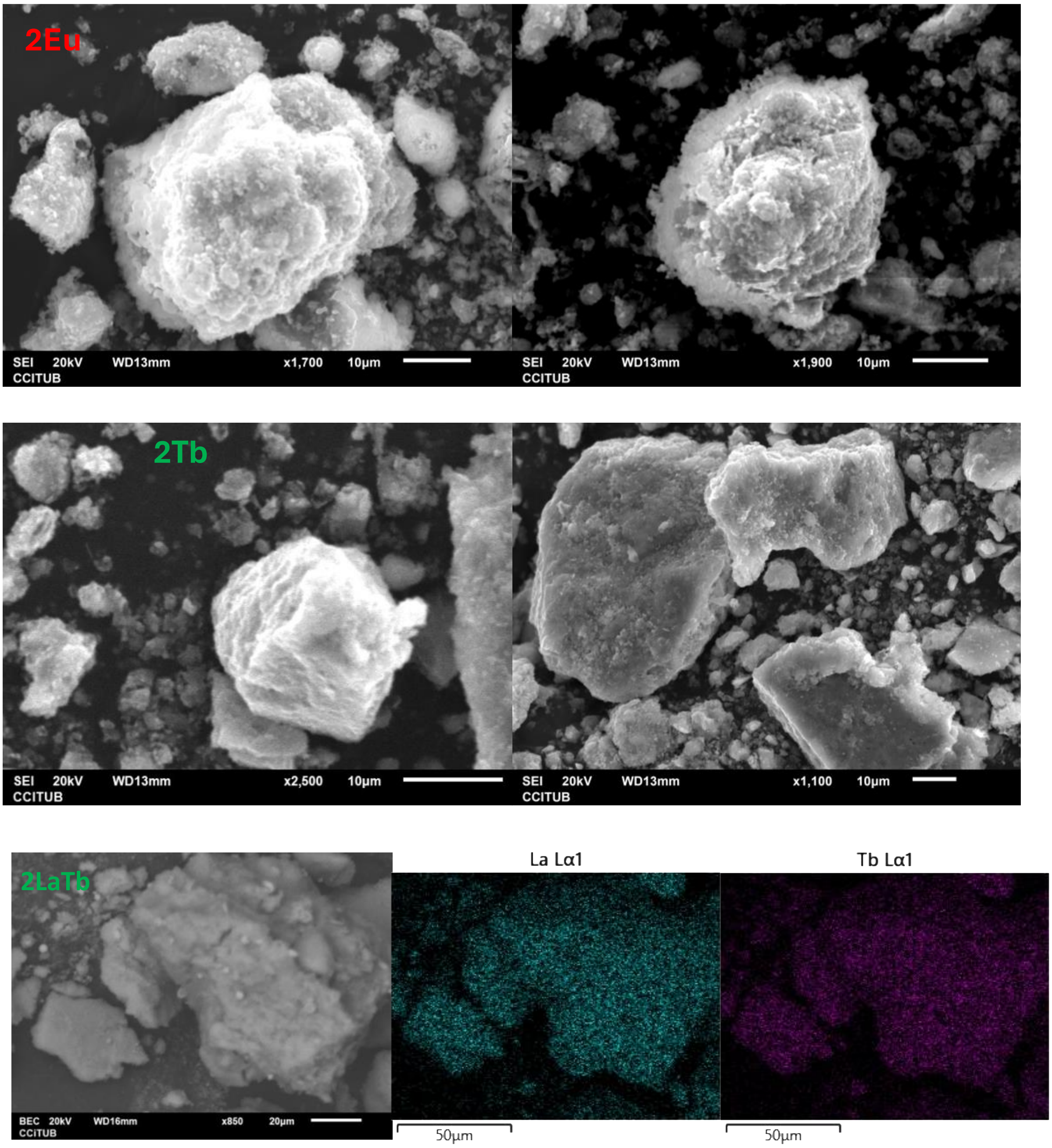

Figure S4. Thermogravimetric analysis (TGA) and differential scanning calorimetry (DSC) for **2Tb** and **2Eu**.

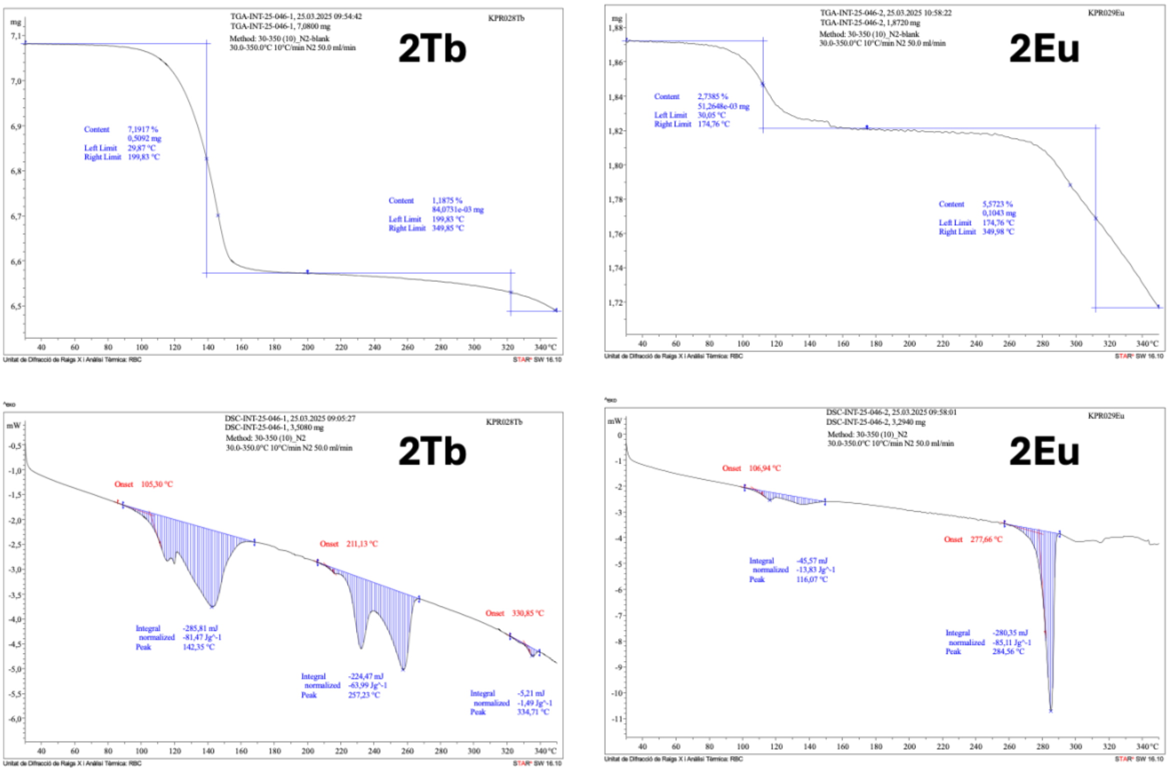

Supplement: Supplementary file 1 [file ic5c01592_si_001.pdf]
